# Supplementary figures and images for: Congenital Rift Valley fever in Sprague Dawley rats is associated with diffuse infection and pathology of the placenta
Source: PLoS Negl Trop Dis. 2022 Oct 31;16(10):e0010898. doi: 10.1371/journal.pntd.0010898 (PMC9648853; doi:10.1371/journal.pntd.0010898)

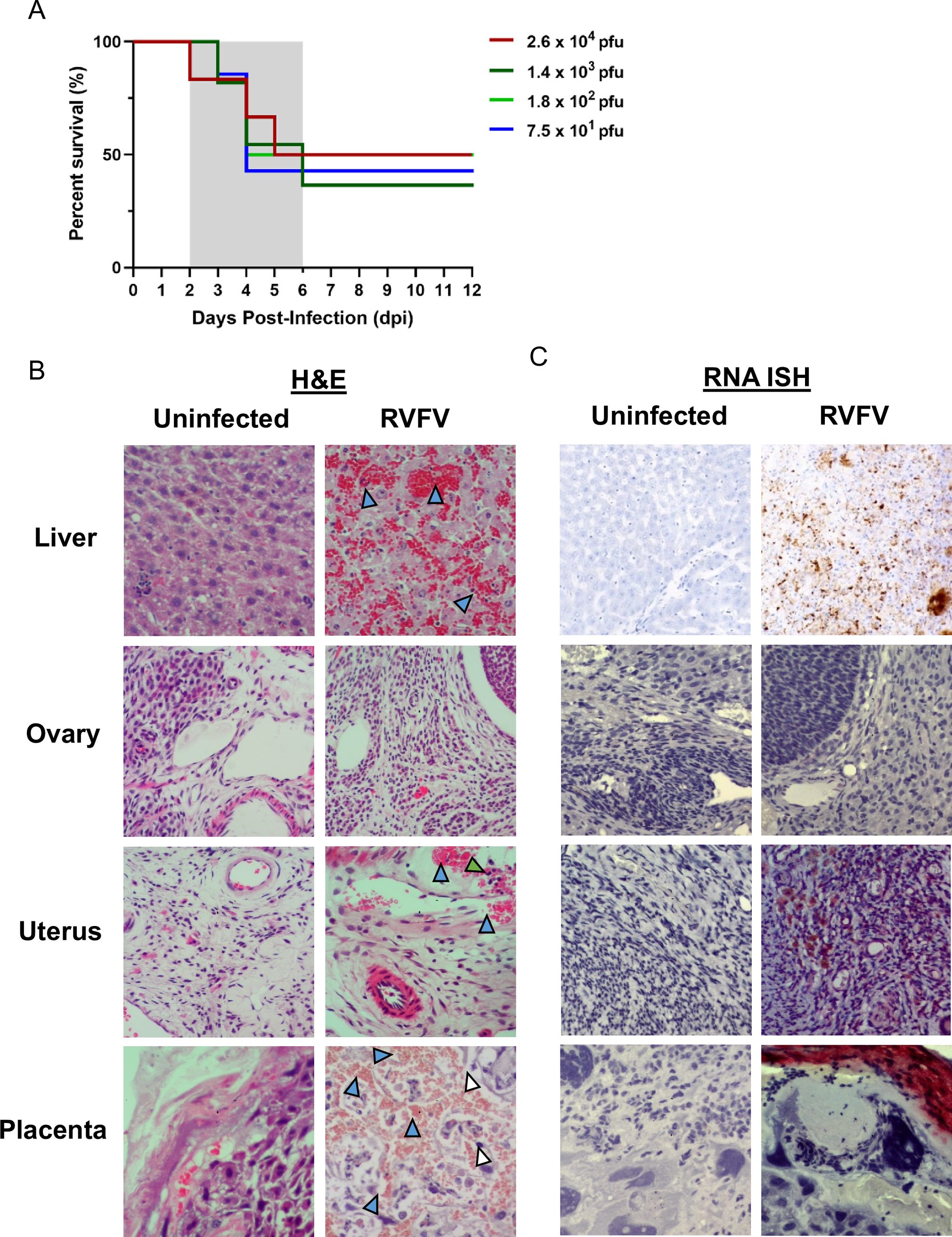

Supplement: S1 Fig — (A) Survival of SD rats infected at E14. Pregnant rats were infected with the indicated doses of RVFV (1.5x10^5^ pfu (n = 3), 2.6x10^4^ pfu (n = 6), 1.4x10^3^ pfu (n = 11), 1.8x10^2^ pfu (n = 6), 75 pfu (n = 1)). The grey shaded area between 2-6dpi indicates the clinical window, when lethally infected dams were euthanized due to severe disease. (B) H&E and (C) RNA ISH staining (brown/magenta) for viral RNA within the liver, ovary, uterus, and placenta from uninfected and RVFV infected dams who met euthanasia criteria. Blue, white, and green arrow heads indicate hemorrhaging, necrosis, or leukocyte inflammation, respectively. Hematoxylin counterstain. (TIF) [file pntd.0010898.s001.tif]

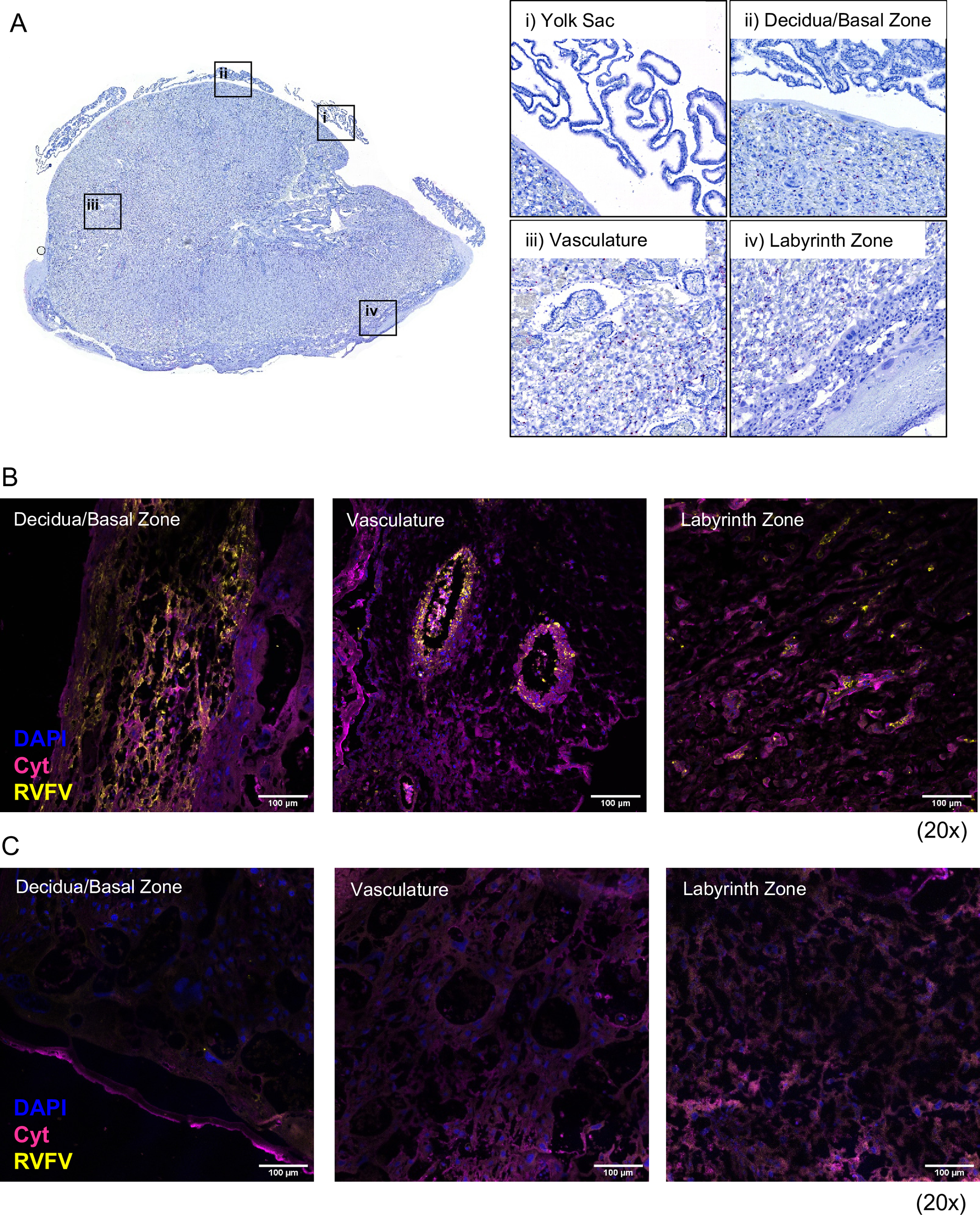

Supplement: S2 Fig — (A) Left panel: cross-section of an uninfected control placenta (E20) with ISH for RVFV viral RNA (magenta). Hematoxylin counterstained. The following structures were imaged for the right panels: (i) yolk sac, (ii) decidua and basal zone, (iii) maternal vasculature, and (iv) labyrinth zone. (B) RVFV-infected or (C) uninfected placentas at E20 were stained with anti-RVFV Gn antibodies (yellow), anti-cytokeratin antibodies (magenta), and DAPI followed by immunofluorescent microscopy. The following structures were imaged: decidua and basal zone (left), maternal vasculature (middle), and labyrinth zone (right). 20x images. (TIF) [file pntd.0010898.s002.tif]

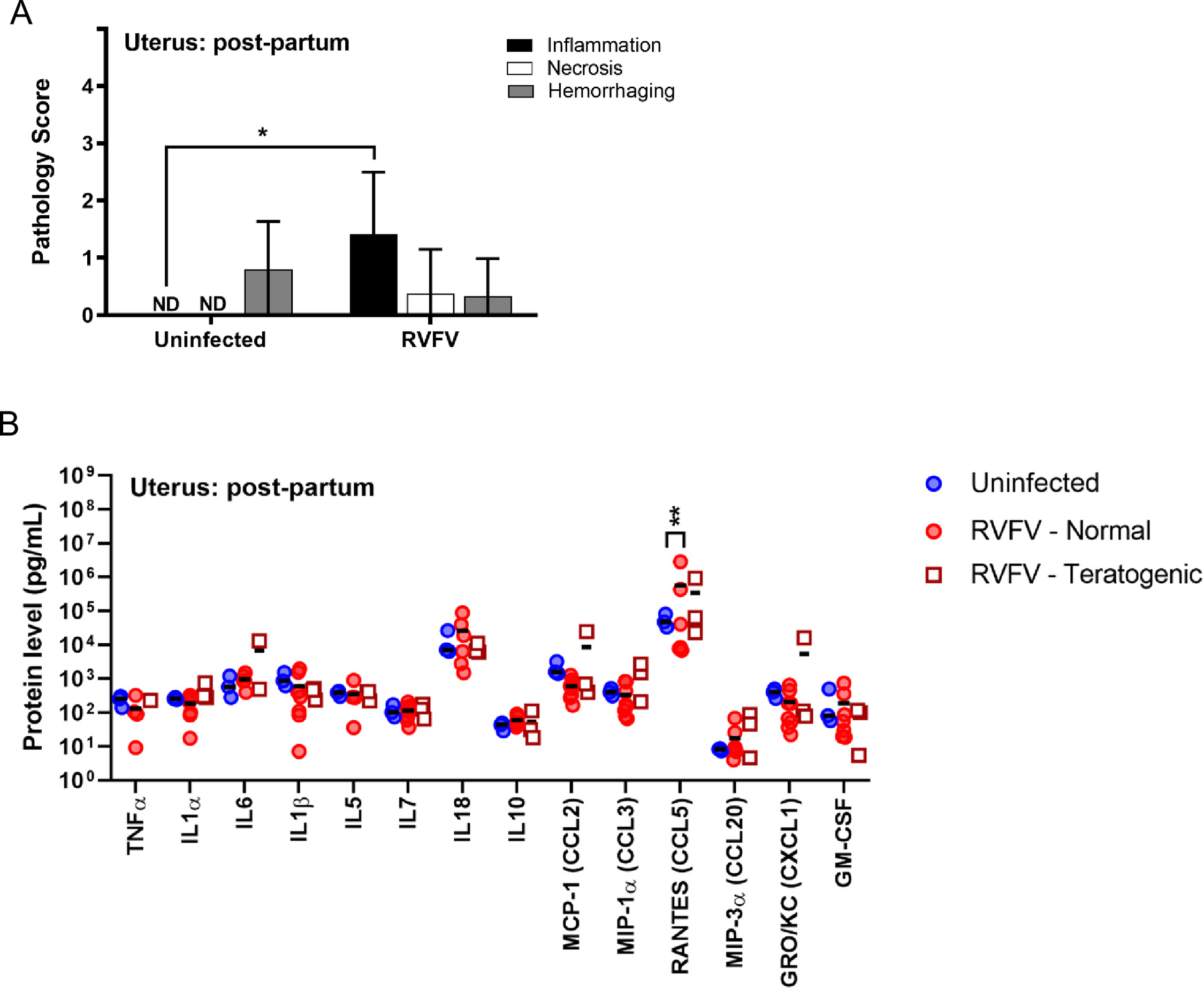

Supplement: S3 Fig — (A) Pathology scores identifying inflammation, necrosis and hemorrhage severity of H&E stained uterus of RVFV infected (n = 12) or uninfected dams (n = 5) that survived to post-partum (euthanized 18-22dpi). (B) Protein levels (pg/mL) of cytokines, chemokines, and growth factors within the uterus of infected (RVFV–Normal (n = 4–8), RVFV -Teratogenic (n = 2–4) and uninfected (n = 3) dams that survived to post-partum. * = p <0.05, ** = p < 0.01. ND = none detected. An ANOVA with multiple comparisons was performed to determine statistical significance between the cohorts. (TIF) [file pntd.0010898.s003.tif]

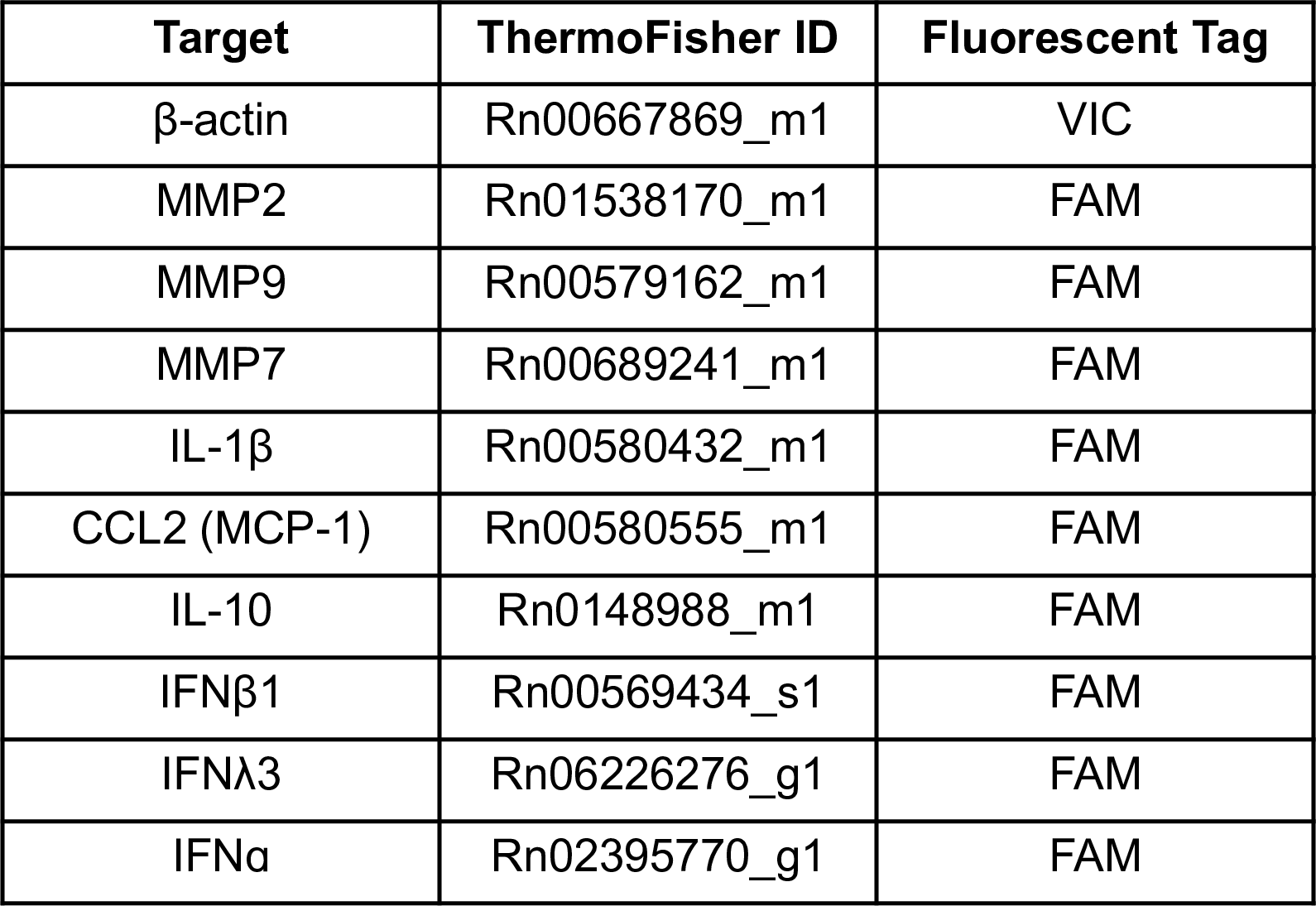

Supplement: S1 Table — (TIF) [file pntd.0010898.s004.tif]
