# Supplementary material for: Congenital Rift Valley fever in Sprague Dawley rats is associated with diffuse infection and pathology of the placenta
Source: PLoS Negl Trop Dis. 2022 Oct 31;16(10):e0010898. doi: 10.1371/journal.pntd.0010898 (PMC9648853; doi:10.1371/journal.pntd.0010898)
Supplement: Attachment — Submitted filename: Response to reviewers comments FINAL v2.docx [file pntd.0010898.s005.docx]

**We thank the reviewers for their careful reading of our manuscript and helpful suggestions. Our responses to the reviewers’ comments are listed below in** **bolded blue.**

**Methods**

Reviewer #1: More detail for euthanasia criteria/scoring (or a reference to previously published work that lists in more detail) would be helpful (weight loss cut-offs, etc). In particular criteria that tend to be met most often for this model in their hands.

**These details have been added to the methods section starting at line 566. A table was also included for clarification.**

**Results**

Reviewer #2: I have only two comments.

1) Throughout the manuscript the authors chose to use "Viral titer" when the results look striking and larger (e.g., line 153) and just "viral RNA levels" (e.g., line 159)... At all times from what this reviewer can discern the author utilize RT-qPCR for viral RNA detection and presumably quantitation. This is NOT a "viral titer". I think to keep things clear the authors should revert to some other nomenclature where this is more obvious. I am NOT suggestion to go back and do live-virus titrations of all the tissues... but please clear up the language so that it is obvious you are talking about viral RNA and not infectious virus. **We thank the reviewer for pointing this out. This was corrected to “viral RNA levels” or similar vocabulary on lines 137, 157, and 158.**

2) Another puzzling observation was the lack of cellular inflammation amongst the placentas examined. The authors relatively down play that observation, but I think it might be actually quite telling regarding the underlying pathophysiological mechanisms leading to placental infection/pathology and eventual pup teratogenesis. Could you please add in a bit more on that section in the discussion. **We agree that it is an interesting observation. A more detailed explanation for the lack of inflammation was included in the conclusions/discussion section starting at line 468.**

**Conclusions**

Reviewer #1: One suggestion is to also note in text the different placental structures (ruminant vs. rodent/human), as there are several instances where the rat is suggested as a model for susceptible ruminants, and while it may apply in certain cases the placental structure differences should be clearly stated as a potential limitation in that particular application. **We appreciate this comment and have included additional descriptions of the differences in structure between rodent, livestock, and human placenta. This can be found in lines 383-400 and lines 421-428.**

Reviewer #2: The authors may want to be a bit more cautious with direct extrapolations from multiple species (rodents and livestock) to humans. While it is clear that RVFV is infecting and causing vertical transmission in these SD rats, the data from actual human placentas and other aborted materials is extremely limited. These studies are likely a key guide marker, but may not directly be applicable to humans. This reviewer recommends at least some discussion of that aspect of this animal model study.

**This point is well taken and we have clarified these limitations on lines 399 & 427.**

Additionally, Line 463: please modify the text from "sacrifices" to "euthanasia"... in experimental animal work we are euthanizing animals when necessary, not sacrificing them upon an altar on a mountaintop. This may seem trivial, but I think euthanasia is more appropriate verbiage for the scientific literature and when talking with non-scientific audiences. **We appreciate this perspective and the change was made on line 510.**

**Editorial and Data Presentation Modifications**

Reviewer #1: Minor suggestions to improve clarity and workflow:

-Line 123 - 126: Please state clearly when sham-infected controls were euthanized. In parallel with animals that met euthanasia criteria? All at end of study? If applicable, add to study design figure or legend. **This information was added on line 126 in the results section and line 575 in the methods section.**

-Figure 1: Add number of animals in groups specified and mention of sham-inoculated.

e.g., Sprague Dawley (SD) rats were inoculated subcutaneously (s.c.) with RVFV ZH501 at embryonic day 14 (E14; n = ?) or sham inoculated (n = ?).

e.g., "Eight days later (E22), the rats delivered their pups and placentas (n = ?) were...."

**Information regarding the number of infected animals is now included in Fig 1 legend. Information regarding uninfected animals is included in the other figure legends where this information is most pertinent.**

-Please revisit all figure legends and add groups sizes where applicable. **This information is now added to all figure legends.**

-consider using sham-inoculated consistently throughout text and figures; NI is not clear to reader if they served as both tissue and handling controls without looking in more detail to methods. **“Sham-inoculated” was removed and changed to uninfected or no infection (NI) for clarification.**

-Line 142: suggest adding info on lethality in model - i.e., ...leading to fulminant hepatic disease "and fatal outcome in xx - xx% of rats" **This information is now added on line 145 & 146.**

-Line 148: should this be "euthanized from 2-6 dpi" vs. 2-5 dpi? **This should stay as 2-5 dpi because we are specifically referring to the fact that teratogenicity was not observed for animals euthanized prior to 6dpi. We added clarification to the text on line 152.**

-Line 154: avg. 1.8 x 10^8 pfu/mL "equivalent" **This is now corrected.**

-Line 218: Please consider use of term "longitudinal" evaluations, this suggests these were predetermined time points with sufficient groups sizes at each time point vs. animals that met end-point criteria at different times. These analyses more accurately look at tropism in the placenta of animals that succumb to RVFV infection (at different times). For same reason, this study design does not necessarily "identify earliest structure targeted by RVFV", but rather structures infected in animals that reach criteria at different time points (some earlier than others). **This is now corrected on lines 221, 241, 251 and we removed the term “earliest” on line 222.**

-Above comment also applies to Figure 4 legend - suggest removing "longitudinal" and just stating "Pathology in infected placentas from dam succumbing to disease from E16-E20... **This is now corrected on line 223.**

-Suggest clarifying in figure headers when analysis is focused solely on end-point tissues...e.g., for figure 3: RVFV infection is detected in the placenta of dams "that succumb to infection" as early as E16 (2dpi) –

**The legends of figures 3 & 4 were corrected to incorporate this recommendation.**

Figure 5/6: both list a range of group sizes per time point which is not helpful as the data is analyzed in aggregates (NI, normal, teratogenic) - can group sizes be given for these instead to align with analyses presented? **This information is now added to figures 5 & 6.**
